# Purification and characterization of a highly specific polyclonal antibody against human extracellular signal-regulated kinase 8 and its detection in lung cancer

**DOI:** 10.1371/journal.pone.0184755

**Published:** 2017-09-13

**Authors:** Na-Li Cai, Andy T. Y. Lau, Fei-Yuan Yu, Dan-Dan Wu, Li-Juan Dai, Hai-Ying Mo, Chang-Min Lin, Yan-Ming Xu

**Affiliations:** 1 Laboratory of Cancer Biology and Epigenetics, Department of Cell Biology and Genetics, Shantou University Medical College, Shantou, Guangdong, P. R. China; 2 Department of Histology and Embryology, Shantou University Medical College, Shantou, Guangdong, P. R. China; University of Hong Kong, HONG KONG

## Abstract

Extracellular signal-regulated kinase 8 (ERK8), proposed as a novel potential therapeutic target for cancer, has been implicated in cell transformation, apoptosis, the protection of genomic integrity, and autophagy. To facilitate ERK8 research, a highly specific anti-ERK8 antibody is needed. In this article, we use the Immune Epitope Database and Analysis Resource online tool to predict B-cell epitopes of human ERK8 protein, and choose a 28 aa-peptide sequence to generate the GST-ERK8(28aa) fusion protein as the antigen for developing polyclonal antibody against ERK8. The specificity and sensitivity of anti-ERK8 antibody were robustly validated by immunoblotting, immunocytochemical and immunohistochemical analyses; and we found that both the endogenous and ectopically-expressed human ERK8 proteins can be recognized by our anti-ERK8 antibody. This suggested that our characterized anti-ERK8 antibody will be a valuable tool for the elucidation of the distribution of ERK8 at cellular and histological levels. Finally, our tissue array analysis also demonstrated that the ERK8 protein was localized in both the nucleus and cytoplasm of human lung cancers.

## Introduction

Mitogen-activated protein kinases (MAPKs) are a super-family of proline-directed serine/threonine kinases that have been implicated in cell proliferation, differentiation, apoptosis, and stress response [1, 2]. ERK8 (alias MAPK15), as one of the most recently identified members in the ERK family, possesses two SH3-binding motifs in its characteristically C-terminal region and can be auto-phosphorylated [3]. It has been reported that serum, DNA damage, H_2_O_2_ treatment, and activated human oncogenes such as BCR/ABL1, RET-MEN2B, and RET/PTC3 can activate ERK8 [4–6]. ERK8 was demonstrated to regulate cell proliferation, transformation, and the protection of genomic integrity [7, 8]. As a regulator of autophagy, ERK8 induces the autophagic process by interacting with LC3 and GABARAP proteins [9]. Moreover, ERK8 can affect telomerase activity and the activity of nuclear receptors [10–12]. Consequently, ERK8 is proposed to be a potential clinical therapeutic target.

Although ERK8 commercial antibodies are available; however, these commercial anti-ERK8 antibodies have low sensitivity and specificity which may hinder the functional research for ERK8. In this study, we report the design, expression, and purification of the recombinant ERK8 fusion protein, i.e., GST-ERK8(28aa), a designated 28 amino acid region of human ERK8 fused to GST and use it as an antigen to generate the anti-ERK8 polyclonal antibody. The polyclonal antibody against human ERK8 was produced by immunizing rabbit with this antigen. The purified antibody exhibited high sensitivity and specificity, which was validated by immunoblotting, immunocytochemical and immunohistochemical analyses. This is the first formal report that detailing the production of polyclonal antibody against ERK8 and the anti-ERK8 antibody thus generated it will enable further investigation into the biological function of ERK8.

## Materials and methods

### Materials

shRNAs were purchased from Santa Cruz Biotechnology (Santa Cruz, CA). ERK8 shRNA plasmid (h) (sc-77462-SH) is a target-specific lentiviral vector plasmid encoding a 19–25 nt (plus hairpin) shRNA to knockdown gene expression. Control shRNA plasmid-A (sc-108060) encodes a scrambled shRNA sequence that will not lead to the specific degradation of any known cellular mRNA. All other general chemicals were purchased from GE Healthcare (Uppsala, Sweden), Qiagen (Valencia, CA) and Sigma-Aldrich (St. Louise, MO). The *Homo sapiens* ERK8 gene coding sequence (NM_139021) expression plasmid pGEX-2T-GST-ERK8 was generously provided by Dr. Mark K. Abe from the University of Chicago (Chicago, IL). Xpress and β-actin antibodies used for western blot was from Invitrogen (46–0528) and Sigma-Aldrich (A5441), respectively. Besides the purified anti-ERK8 antibody generated in our laboratory, four other ERK8 antibodies used for our experiments were purchased from commercial companies.

### Antigen prediction of ERK8

B-cell epitopes are parts of proteins or other molecules which can be used to raise antibodies against a specific protein via immunizing animals. An online bioinformatics tool Immune Epitope Database and Analysis Resource (IEDB, http://www.iedb.org/) [13] was used for predicting B-cell epitopes of human ERK8. Specific immunogenic peptide from 446 to 473 amino acids of ERK8 was selected as polypeptide antigen sequences which is referred to as the recombinant GST-ERK8(28aa) antigen fragment.

### Construction of pGEX-5X-1-ERK8(28aa) plasmid

A 84 bp-sequence from 1336 to 1419 base pairs of human ERK8 DNA coding region was cloned into pGEX-5X-1 plasmid at BamH I and Not I restriction sites to generate a NH_2_-terminal GST-tagged fusion protein. The DNA sequence was amplified by PCR using forward (5′-CGGATCCTTATGCCAAGCGGGAGGGGAGCT-3′) and reverse (5′-TTGCGGCCGCTTACCAGTCACCCCGGAT-3′) primers. Amplification was carried out using the following programs: initial denaturation at 95°C for 3 minutes followed by 25 cycles of denaturation at 95°C for 30 seconds, annealing at 70°C for 30 seconds, extension at 72°C for 30 seconds, and then final extension at 72°C for 7 minutes. Next, PCR product was gel purified and digested with BamH I and Not I restriction enzymes (TaKaRa, Dalian, China), the purified PCR product was then ligated to the BamH I/Not I site of pGEX-5X-1 vector using T4 ligase (TaKaRa) and the ligation mix was transformed into *E*. *coli* strain DH5α. The resulting recombinant plasmid pGEX-5X-1-ERK8(28aa) was confirmed by DNA sequencing and finally transformed into *E*. *coli* strain BL21(DE3)pLysS for recombinant protein expression.

### GST-ERK8(28aa) protein expression and purification

After transforming the pGEX-5X-1-ERK8(28aa) plasmid into *E*. *coli* strain BL21(DE3)pLysS, different induction conditions including concentrations of isopropyl-β-D-thiogalactopyranoside (IPTG), induction temperature and induction time points were tested to optimize the fused GST-ERK8(28aa) protein expression condition. As the final result, GST-ERK8(28aa) fusion protein was induced with 0.1 mM IPTG at 37°C for 8 hours when the optical density (OD_600_) of the *E*. *coli* culture reached 0.6. After expression, the cells were harvested by centrifugation (12,000 rpm, 30 minutes, 4°C), resuspended in 1×PBS, disintegrated by sonication, and incubated with Glutathione Sepharose 4B beads (GE Healthcare) overnight at 4°C. Then the beads were washed with 1×PBS for 6 times. The protein bound to the sepharose beads was eluted using GSH elution buffers (10 mM Glutathione in 50 mM Tris-HCl, pH 8.0), concentrated and GSH removed using Amicon Ultra-4 centrifugal filter units (Millipore). The eluate was then quantified using Bradford protein assay (Bio-Rad) and visualized by Coomassie Blue staining after SDS-PAGE. The authenticity of the purified GST-ERK8(28aa) was further confirmed by trypsin digestion and mass spectrometry (MS) proteomic analysis (Wininnovate Bio, Shenzhen, China).

### Immunization and production of the ERK8-reactive rabbit polyclonal antibody

New Zealand White rabbits were used to produce antibodies against the purified proteins. Prior to immunization, a blood sample was collected intravenously from the ear vein and the serum was used as a negative control for later antibody characterization. For primary immunization, 1 mg protein in Freund’s complete adjuvant (Sigma-Aldrich) was inoculated subcutaneously on the dorsal back of rabbit. After that, the rabbit received two boosters of 500 μg in incomplete Freund’s adjuvant (Sigma-Aldrich) at two-week intervals. The last booster was inoculated intravenously from the ear vein without any adjuvant. One week after the final injection, 60–80 mL of blood was collected and kept overnight at 4°C to allow clotting of the blood. Crude antiserum was collected by centrifugation (4,200 g for 5 minutes) and anti-GST immunoglobulins (IgG) were depleted by incubating the antiserum with GST-bound GSH Sepharose beads overnight.

### Antiserum titer determination by ELISA

Indirect ELISA was used to measure the antibody titer. Purified ERK8 protein was diluted to 10 ng/μL in 50 mM carbonate-bicarbonate buffer (pH 9.6), then coated on each well at 50 μL aliquot per well in a 96-well ELISA plate at 4°C for 12 hours. After washing three times with PBS (pH 7.4), 200 μL of 5% BSA was added to each well and incubated at room temperature for 2 hours. After blocking, 100 μL of serial diluted anti-ERK8 serum (1:400 to 1:819,200) was then added into each well of the plate. After incubation at room temperature for 2 hours and washed with PBS for four times, the wells were incubated with horseradish peroxidase-conjugated goat anti-rabbit IgG (Sigma) (dilution 1:10,000) for 2 hours at room temperature. Peroxidase activity was detected using 3,3′,5,5′-tetramethylbenzidine (TMB) and H_2_O_2_ as substrates. After color development for 25 minutes, 2 M of H_2_SO_4_ stop solution was added to each well and the absorbance was measured at 450 nm using a microplate reader (Thermo Scientific).

### Cell culture and transfection

All the cell lines employed in this study, including HCT15, HCT116, 293T, H460, Calu-3, H1299, H358, and HeLa cells were purchased from and authenticated by the ATCC Cell Bank of the Chinese Academy of Sciences (Shanghai, China) and cultured following the supplier’s recommended conditions. Transfection of the expression vectors was performed using Lipofectamine (Invitrogen) following the manufacturer’s suggested protocol. For generation of ERK8 knockdown cells, viral particles were packed with control or ERK8 shRNA. H460 cells, grown to about 70% confluence, were infected with the above lentiviral shRNAs in the presence of 8 μg/ml polybrene for 24 hours. Uninfected cells were eliminated by exposure to 2 μg/ml puromycin for 4 days before use.

### Quantitative real-time RT-PCR

In brief, total RNA was extracted from cells using TRIzol Reagent (Thermo Fisher Scientific, 15596018). cDNA was synthesized using PrimeScript Reverse Transcriptase (TaKaRa, 2680A) according to the manufacturer's instructions. Quantitative real-time RT-PCR was performed with GoTaq qPCR Master Mix (A6001) from Promega (Fitchburg, WI) and gene-specific primers on Applied Biosystems 7500 Real-Time PCR System. β-Actin was amplified as internal reference to normalize gene expression. Primers were synthesized by Beijing Genomics Institute with the following sequences: ERK8 (forward: 5′-GACCAGAAGCCGTCCAATGT-3′, reverse: 5′-GTATCGGTGCGAAGAGAGCA-3′) and β-actin (forward: 5′-ATGGGTCAGAAGGATTCCTATGTG-3′, reverse: 5′-CTTCATGAGGTAGTCAGTCAGGTC-3′).

### Western blot analysis

Total cell lysate was attained by adding cells with RIPA buffer followed by sonication and centrifugation. The clarified supernatant was used as total cell lysate. Proteins was separated by appropriate percentage of SDS-PAGE and transferred to a PVDF membrane. Membranes were blocked with 5% non-fat milk (w/v) in PBS 0.05% Tween-20 (PBS-T) for 90 minutes at room temperature and incubated with primary antibody overnight at 4°C. Then the membranes were washed with PBS-T and incubated with secondary antibody for 120 minutes at room temperature. After the membranes were washed, the immunoreactive bands were detected using enhanced chemiluminescence (ECL) reagents.

### Immunocytochemical analysis

24 hours after transfection with pcDNA4-Xpress-ERK8, HeLa cells were fixed with 4% paraformaldehyde at room temperature for 10 minutes and permeabilized with 0.2% Tween-20 at room temperature for 20 minutes. Cells were then blocked in 5% goat serum for 1 hour at room temperature, ERK8 was detected by using mouse anti-Xpress primary antibody (Life technologies; 1:200), and also by our purified rabbit anti-ERK8 antibody (1:250) or four other commercial ERK8 antibodies (1:250), followed by incubation with Texas Red-labeled anti-mouse IgG secondary antibody (sc-2781; Santa Cruz biotechnology), 1:100; and FITC-labeled anti-rabbit IgG secondary antibody (sc-2012; Santa Cruz biotechnology), 1:100. Finally, Fluoro-gel with DAPI (Emsdiasum, Hatfield, PA) was used to visualize the nucleus, and the cells were examined on an Axiovert 40 CPL fluorescence microscope.

### Immunohistochemical analysis

Lung cancer and normal lung tissue array were used for immunohistochemical staining of ERK8 protein with our antibody against ERK8. The tissue sections were deparaffinized with xylene and rehydrated with gradient concentrations of ethanol. For quenching the endogenous hydrogen peroxidase, the sections were immersed in 3% H_2_O_2_ for 40 minutes. Antigen retrieval was carried out by incubating the sections with 0.01M citrate buffer and keep heating for 20 minutes. Upon cooling down naturally, the sections were washed with 0.01M PBS 5 minutes for 3 times. Non-specific adsorption was blocked in 5% BSA for 1 hour at room temperature. Then the sections were incubated with our home-made anti-ERK8 rabbit polyclonal antibody (1:200) overnight at 4°C. After washing with 0.01 M PBS 5 minutes for 3 times, the sections were incubated with an HRP-conjugated goat anti-rabbit IgG secondary antibody (1:500) at room temperature for 45 minutes. The immunoreactions were performed using the DAB substrate Kit (ZSGB-BIO), then the sections were counterstained lightly with hematoxylin and scanned using a digital microscope scanning platform.

### Blocking peptide assay

A 28 aa-peptide (from region 446 to 473 of human ERK8) (manufactured by Sangon Biotech, China) was used as blocking peptide for western blot analyses. In order to visualize the inhibitory effect of the peptide for the antibody, peptides in 20×, 50×, 100×, and 200× excess compared to antibody molarity were used. Peptides were mixed with the antibody before incubating with PVDF membranes from western blot transfer.

### Statistical analysis

Statistical analysis was done by using two-tailed Student’s *t*-test. A *P* value of < 0.05 was considered significant. All quantitative results were expressed as the mean ± SD of triplicate samples. The reproducibility of experimental results was confirmed in three separate experiments and representative data set was shown.

## Results

### ERK8 sequence analysis and protein epitope prediction

**Fig 1:**
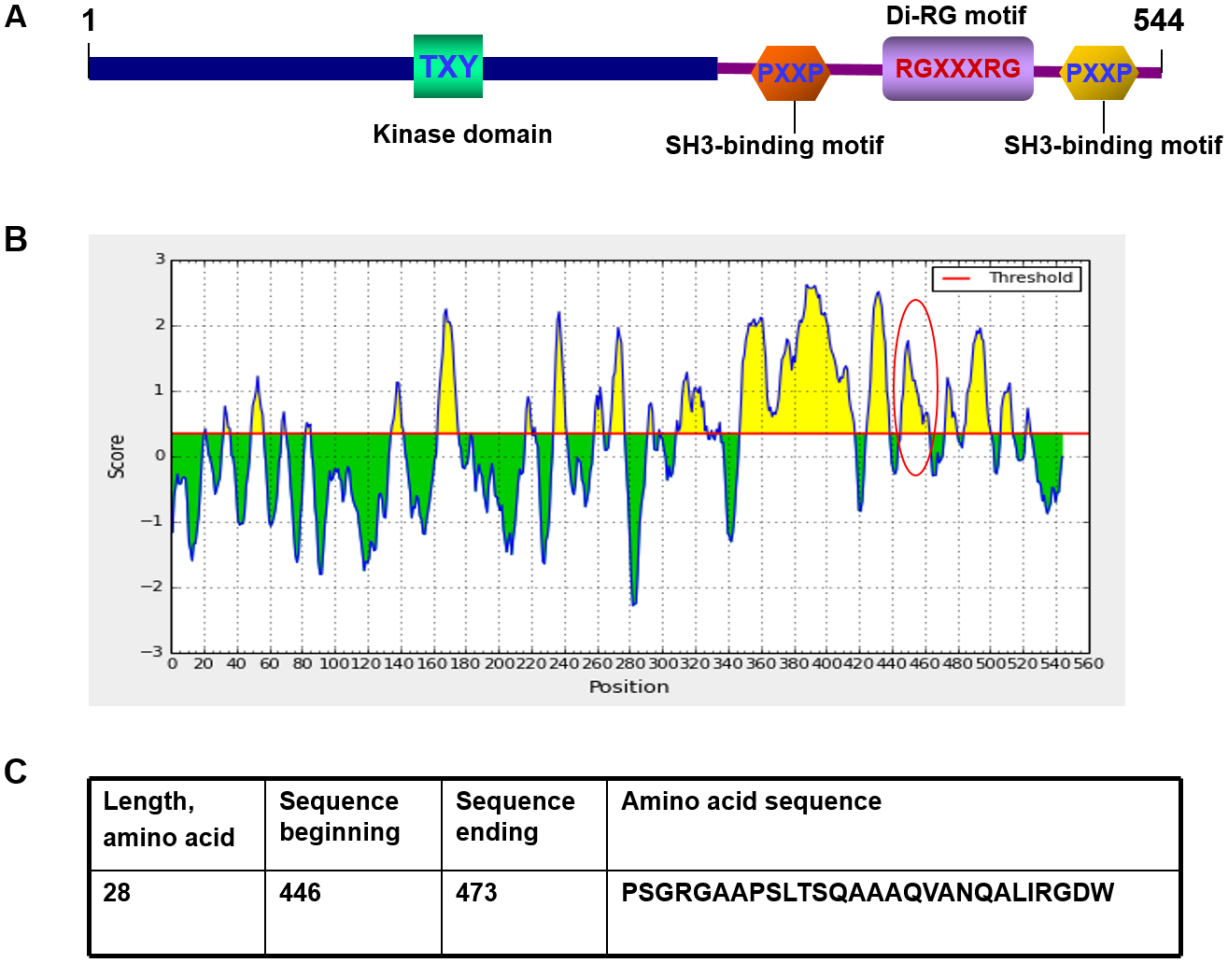
Domain structure and immunogenicity prediction of human ERK8. (A) Domain structure of ERK8. Human ERK8 contains a kinase domain, two SH3-binding motifs, and a di-RG motif. (B) B-cell epitopes of ERK8 was predicted by an online bioinformatics server. X axis and Y axis represent ERK8 protein sequence and the immunogenicity score, respectively. A higher score means the region is more likely to stimulate strong immune response in animals. The encircled peak indicates the amino acid region that successfully producing the home-made polyclonal anti-ERK8 antibody. (C) The amino acid sequence of ERK8 fragment that was chosen for rabbit immunization.

ERK8 is a multi-domain protein that contains the TXY activation motif within the kinase domain, two SH3-binding motifs (PXXP) and a di-RG motif (RGXXXRG) (Fig 1A). To develop an antibody specifically recognizing ERK8 protein, we predicted the B-cell epitopes of human ERK8 using an online bioinformatics server Immune Epitope Database and Analysis Resource. The result demonstrated that the C-terminus of human ERK8 is the more likely region to induce strong immune reaction (Fig 1B). Based on the prediction result, a 28 amino acid-peptide (at position 446–473) of ERK8 was selected as the immunogen (Fig 1C).

### Construction of plasmid, expression, and purification of GST-ERK8(28aa) fusion protein

**Fig 2:**
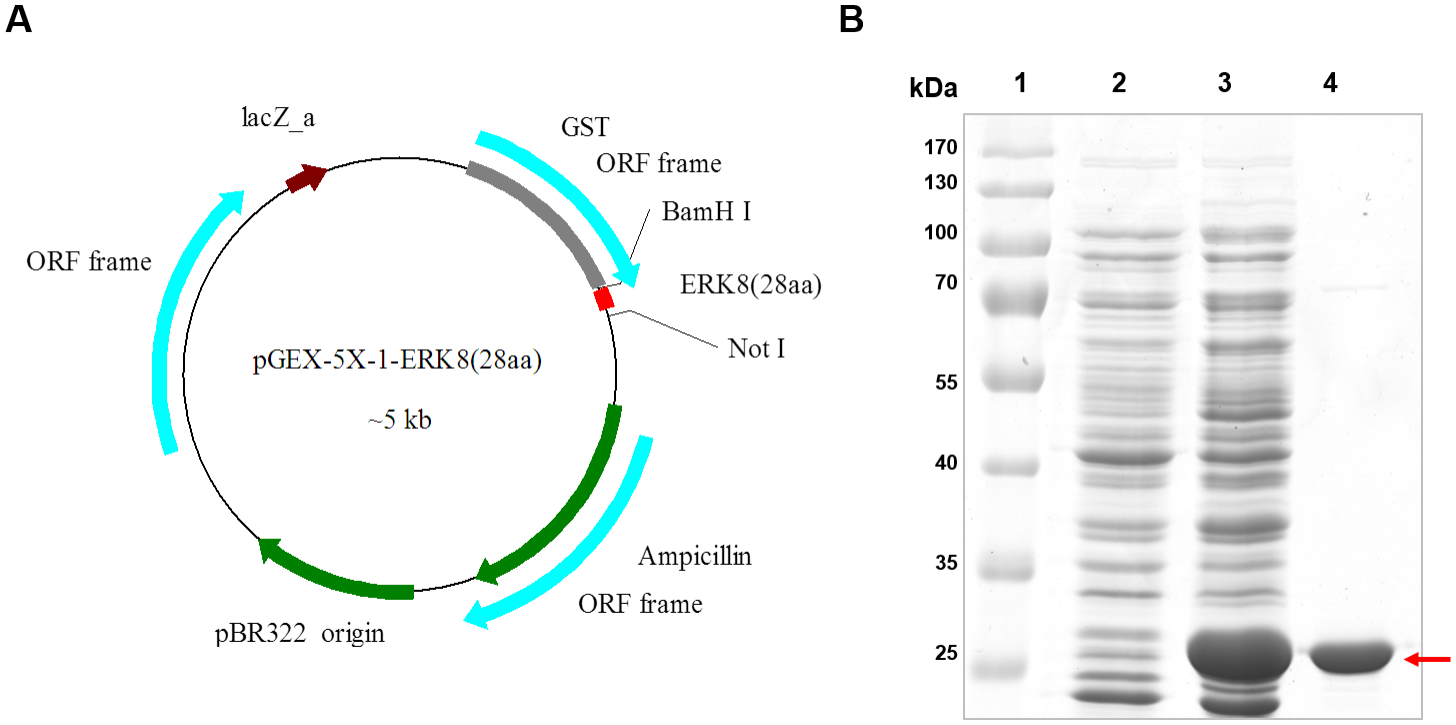
Schematic diagram of the recombinant plasmid pGEX-5X-1-ERK8(28aa) and the purity of the purified GST-ERK8(28aa) fusion protein. (A) Construction of the recombinant plasmid pGEX-5X-1-ERK8(28aa) which contains a GST-tag. (B) GST-ERK8(28aa) fusion protein was highly purified by affinity chromatography. A band indicated by red arrow which could predominantly be seen in the SDS—PAGE gel stained with Coomassie Blue was the recombinant GST-ERK8(28aa) protein. Lane 1: protein ladder; Lane 2: non-induced sample; Lane 3: IPTG-induced sample; Lane 4: purified sample.

For facilitating antigen purification, a GST-tag was fused to the N-terminus of ERK8(28aa) fragment by inserting the amplified immunogen DNA sequence to the pGEX-5X-1 plasmid (Fig 2A). The pGEX-5X-1-ERK8(28aa) plasmid was transformed into *E*. *coli* protein production strain BL21(DE3)pLysS. IPTG was used to induce the expression of the GST-ERK8(28aa) fusion protein. We found that the GST-ERK8(28aa) fusion protein was rapidly and highly expressed in *E*. *coli* as expected. After the GST-ERK8(28aa) was purified from the soluble fraction of the bacterial lysate using Glutathione Sepharose 4B beads, a band of the recombinant GST-fusion protein could predominantly be seen in the SDS-PAGE gel stained with Coomassie Blue (Fig 2B). The authenticity of the purified GST-ERK8(28aa) was further confirmed by MS proteomic analysis, results indicated that the excised band is indeed the GST-fusion protein containing ERK8(28aa) (S1 Fig), suggesting that the GST-ERK8(28aa) fusion protein was successfully purified. Thus, we use it as an antigen for immunization.

### Preparation of polyclonal antibody against ERK8 and the determination of its titer

**Fig 3:**
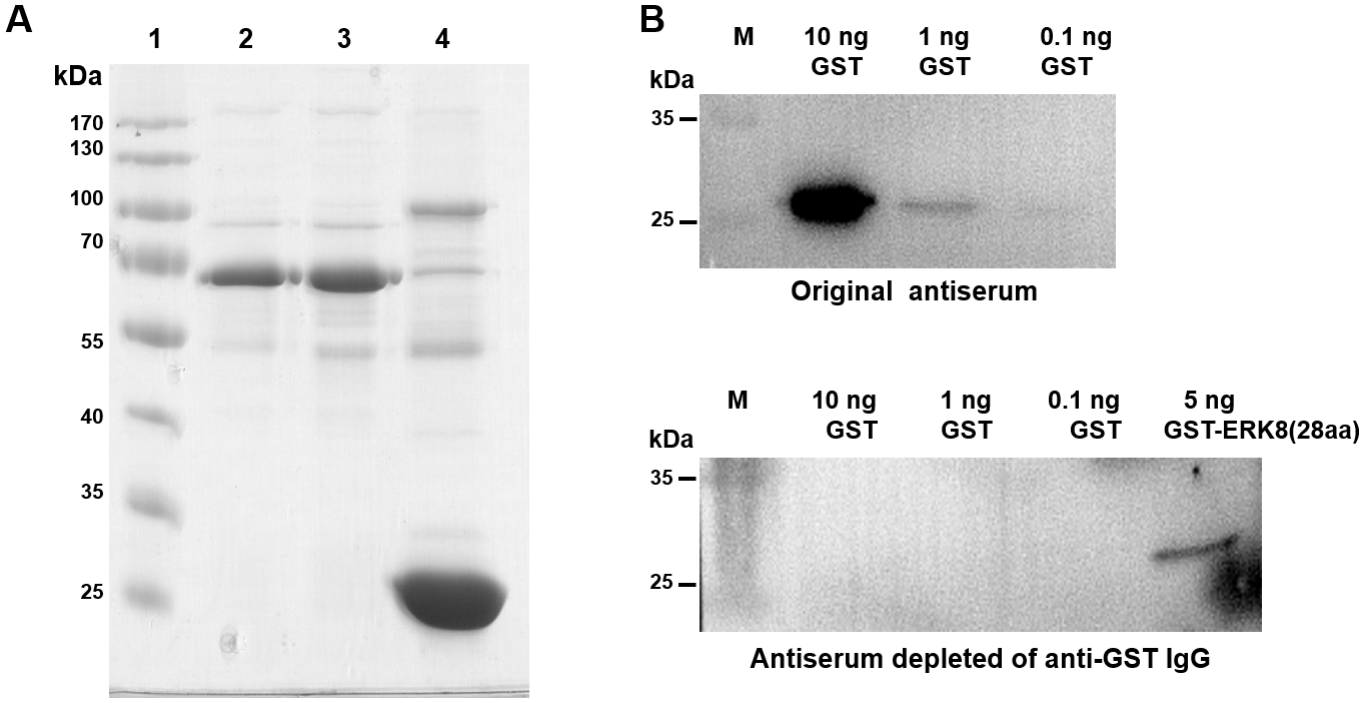
Purification of the antiserum. (A) Anti-GST IgG depletion from the original antiserum analyzed by SDS-PAGE and Coomassie Blue staining. Lane 1: protein ladder; Lane 2: original antiserum; Lane 3: antiserum depleted of anti-GST IgG; Lane 4: GST-bound GSH Sepharose beads incubating with original antiserum. (B) Depletion of anti-GST IgG from the antiserum was analyzed by immunoblots. Original antiserum and purified antiserum were respectively used to probe with purified GST protein. The purified GST-ERK8(28aa) fusion protein was used as a positive control.

**Fig 4:**
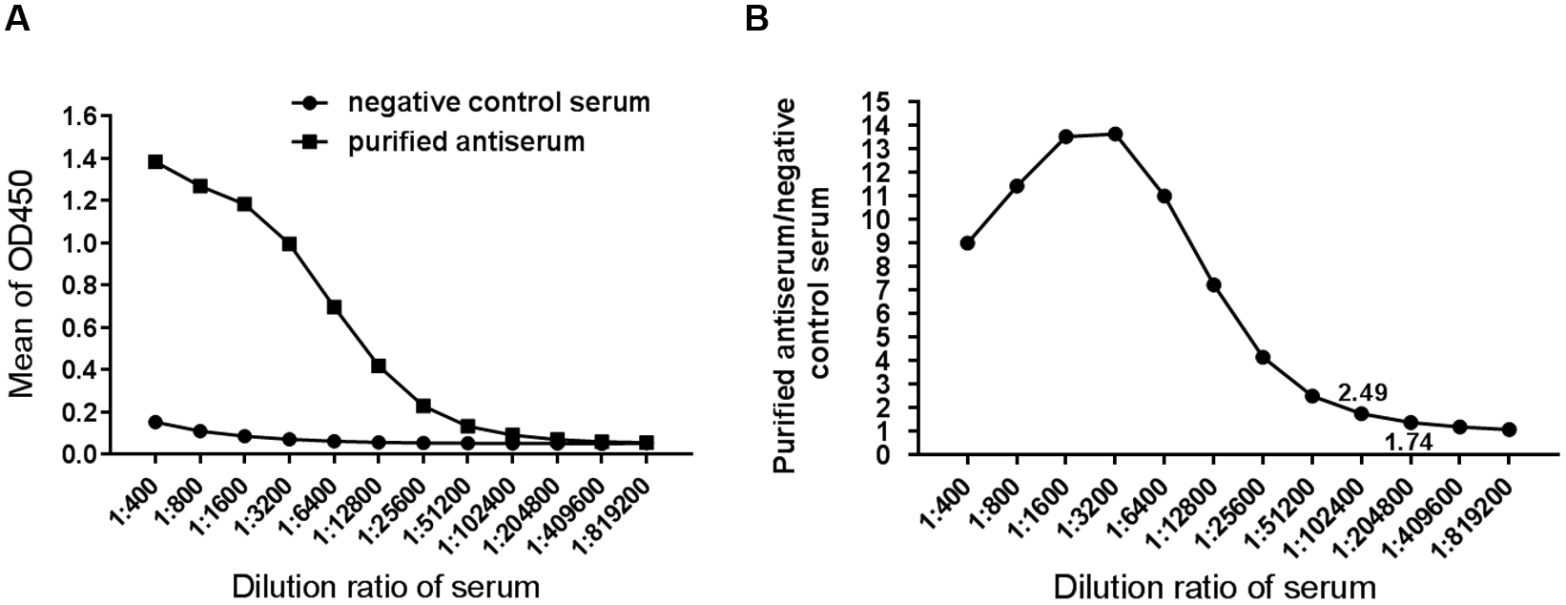
Antiserum titer determination by ELISA. (A) The purified antibody was diluted by serial dilution methods (from 400- to 819,200-fold) and reacted with purified ERK8 protein. Pre-immunized rabbit serum was used as a negative control. (B) The absorbance value ratio of anti-ERK8 antiserum to negative control serum. When the dilution ratio of 1:51,200, the absorbance value ratio of anti-ERK8 to negative serum is 2.49.

The purified GST-ERK8(28aa) protein was used as an antigen to generate polyclonal antibody against human ERK8. After the New Zealand White rabbits were immunized four times with the purified protein, whole blood was collected. Then the antiserum was further purified by GST-bound GSH Sepharose beads since the antigen was fused with a GST-tag, so, depletion of anti-GST IgG was necessary. As shown in Fig 3A, after incubating with the GST-bound GSH Sepharose beads, anti-GST IgG (heavy chain ~50 kDa, light chain ~25 kDa) was depleted from the crude antiserum. To test whether the purified anti-ERK8 antibody is devoided of anti-GST IgG, we performed western blot analyses using various amounts of GST protein (from 0.1, 1, to 10 ng). As expected, the original serum reacted strongly with GST (Fig 3B, upper panel), however, all the signals were abolished when the antiserum was depleted of anti-GST IgG, while only the lane loaded with positive control GST-ERK8(28aa) showed positive signal (Fig 3B, lower panel). These results suggested that the purified antiserum has high immunoreactivity against ERK8(28aa) but not GST protein at all. With indirect ELISA, the negative control serum and purified anti-ERK8 antiserum were assayed at various dilutions for reactivity with ERK8 protein (Fig 4A). Based on the ELISA data, the titer of our resulting polyclonal antibody against ERK8 was estimated to be around 51,200, which indicated that the purified polyclonal antibody has good sensitivity against ERK8 (Fig 4B).

### Determination of the specificity and effectiveness of the purified anti-ERK8 antiserum

**Fig 5:**
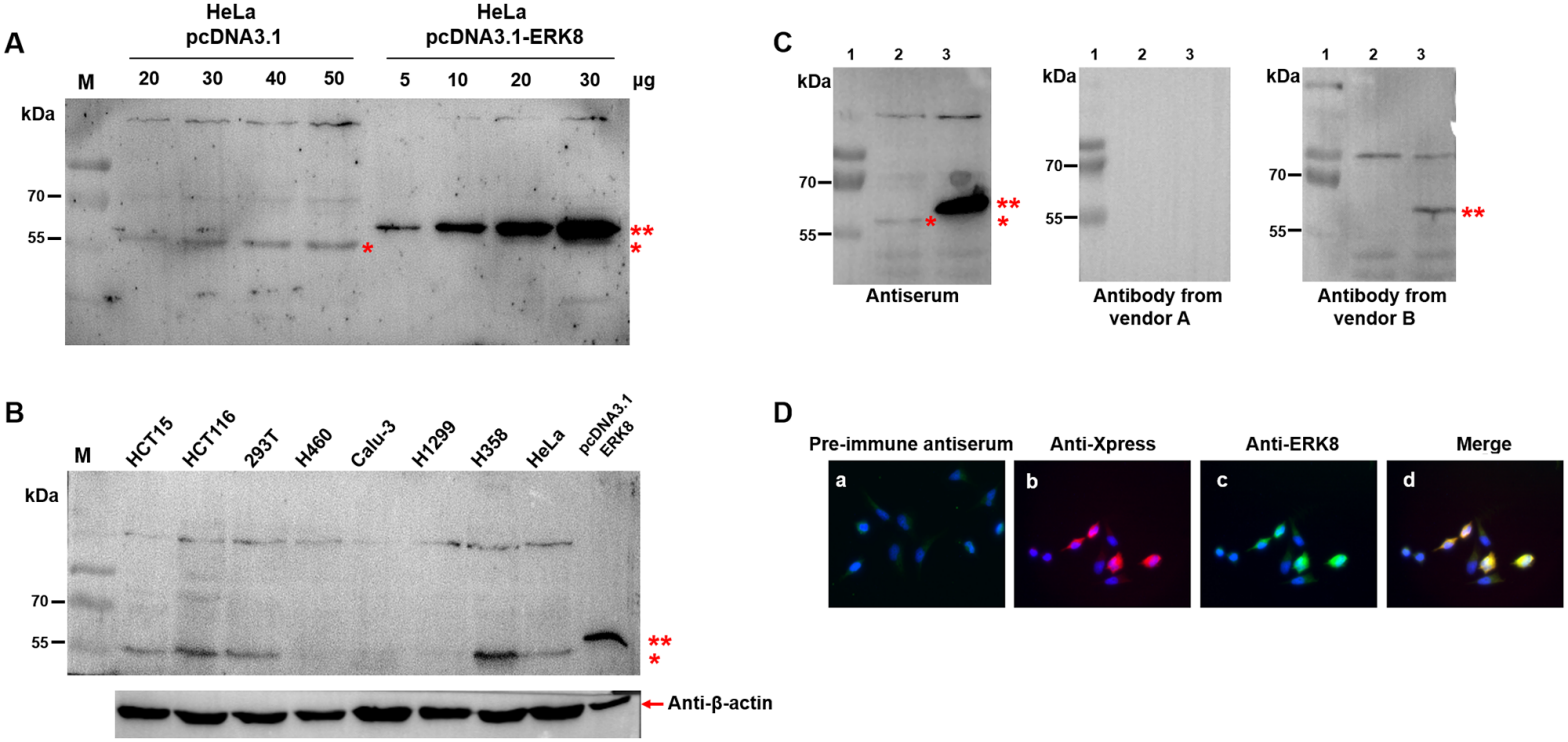
Specificity and sensitivity of polyclonal anti-ERK8 antibody recognizing the endogenous and ectopically-expressed ERK8 proteins. (A) Immunodetection of transient-transfected pcDNA3.1 or pcDNA3.1-ERK8 plasmid in HeLa cells with purified anti-ERK8 serum. The corresponding loading of cell lysates (µg) were also indicated. (B) Immunodetection of endogenous ERK8 expression in HCT15, HCT116, 293T, H460, Calu-3, H1299, H358, and HeLa cells. Expression of ERK8 protein in 293T cells with transfection of pcDNA3.1-ERK8 construct was used as a positive control. β-Actin served as loading control. (C) The efficacy of purified anti-ERK8 serum compared with two commercial ERK8 antibodies (to avoid the conflict of interest, their names were not labeled explicitly). Lane 1: protein ladder; Lane 2: HeLa cells transfected with pcDNA3.1; Lane 3: HeLa cells transfected with pcDNA3.1-ERK8. Single asterisk indicates endogenous ERK8 while double asterisk indicates ectopically-expressed ERK8. (D) Co-immunostaining of transient-transfected Xpress-ERK8 in HeLa cells detected with anti-Xpress antibody (red) and anti-ERK8 serum (green).

To evaluate the specificity of the purified anti-ERK8 antiserum, pcDNA3.1 blank vector and pcDNA3.1-ERK8 plasmid were transfected into HeLa cells respectively and western blot analyses were performed. As shown in Fig 5A, a single band corresponding to endogenous human ERK8 was detected in pcDNA3.1 transfected cells using our purified anti-ERK8 antiserum. In addition, anti-ERK8 antiserum could recognize both the endogenous and ectopically-expressed ERK8 protein in pcDNA3.1-ERK8 transfected cells (ectopically-expressed ERK8 has the V5- and His-tags, therefore, there is a slightly increase in molecular size and thus it migrates slower than the endogenous ERK8) (Fig 5A). Using a panel of human cancer cell lines, a single band at ~55 kDa (endogenous ERK8 protein) was commonly detected in HCT15, HCT116, 293T, H358, and also HeLa cells (Fig 5B). Besides, the efficacy of our purified anti-ERK8 serum was compared with two other commercial ERK8 antibodies (vendors A and B) at the same condition. The results indicated that our purified anti-ERK8 serum could recognize both the endogenous and ectopically-expressed ERK8 proteins with negligible background signals whereas the other two commercial ERK8 antibodies have much weaker (vendor B) or no reactivity (vendor A) with ERK8 protein (Fig 5C). Next, immunofluorescence assay was performed using the pcDNA4-Xpress-ERK8 transfected cells, the results indicated that molecules labeled by the anti-ERK8 antibody had the same localization as Xpress (Fig 5D). However, no immunofluorescence signals were detected using the pre-immune antiserum. Moreover, besides vendors A and B, two more commercial ERK8 antibodies (vendors C and D) were used to compare with our purified anti-ERK8 antibody by immunofluorescence assay (S2 Fig). As just mentioned, the anti-ERK8 antibody from vendor A has no reactivity with ERK8 in western blot (Fig 5C). Although the anti-ERK8 antibody from vendor A can detect ERK8 in immunofluorescence assay, however, the results showed that our home-made antibody has better performance than that of vendor A. Furthermore, all the other three commercial ERK8 antibodies from vendors B, C, and D have very weak immunofluorescence signals, which indicated that they might not be applicable for immunofluorescence assay. To confirm that only ERK8 but not other protein can co-localize with Xpress, here, the pcDNA4-Xpress-RSK2 plasmid (previously constructed by Cho et al. [14]) or pcDNA4-Xpress-ERK8 plasmid was transient-transfected into HeLa cells. As shown in S3 Fig, only ERK8, but not RSK2 signal, co-localized with Xpress signal.

**Fig 6:**
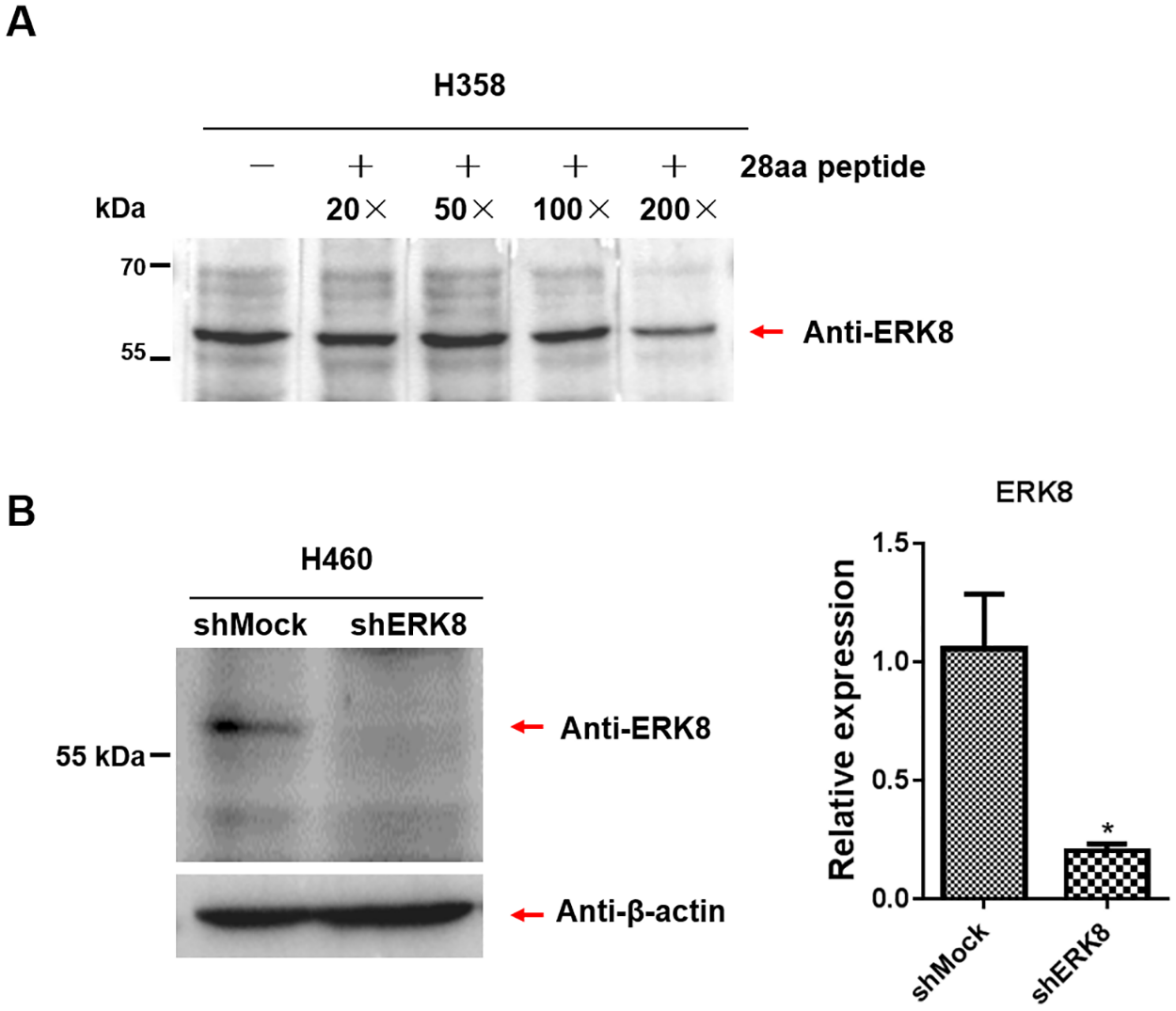
Verification of polyclonal antibody against ERK8. (A) Blocking peptide assay. The ERK8(28aa) peptides in 20×, 50×, 100×, and 200× excess compared to antibody molarity were used to block the binding of our purified home-made anti-ERK8 antibody to its target by western blot analyses using cell lysates from H358 cells. (B) Left panel: ERK8 was knockdowned in H460 cells by viral infection of shERK8 plasmid into H460 cells. H460 shMock and shERK8 cell lysates were subjected to western blot analyses using our purified home-made anti-ERK8 antibody. β-Actin served as loading control. Right panel: ERK8 mRNA level of H460 shMock and shERK8 cells were detected by RT-qPCR. Asterisk indicates a significant difference of *P* < 0.05. The data are representative of three independent experiments.

To further confirm the specificity and effectiveness of the generated anti-ERK8 serum, we used a synthetic ERK8(28 aa) peptide (manufactured by Sangon Biotech) for blocking peptide experiment. As shown in Fig 6A, the blocking peptide was able to disrupt antibody binding to its target. There was a gradual reduction of immune reaction between endogenous ERK8 protein from H358 cells and the anti-ERK8 antiserum incubating with increasing amounts of blocking peptides (more obvious reduction at 100× and 200× excess blocking peptide). Using stable ERK8 knockdown H460 cells, western blot results showed that the purified ERK8 antiserum could not detect any ERK8 signal after knocking down of ERK8 (Fig 6B, left panel). In parallel, the efficiency of knockdown has been validated by RT-qPCR analyses (Fig 6B, right panel). Both results are in agreement with each other in which the level of ERK8 is significantly suppressed at mRNA and protein levels in shERK8 H460 cells. All these data support that our home-made anti-ERK8 antibody can specifically recognize ERK8 at both native and denatured states.

### Detection of ERK8 in human lung tissues using the polyclonal anti-ERK8 antibody

**Fig 7:**
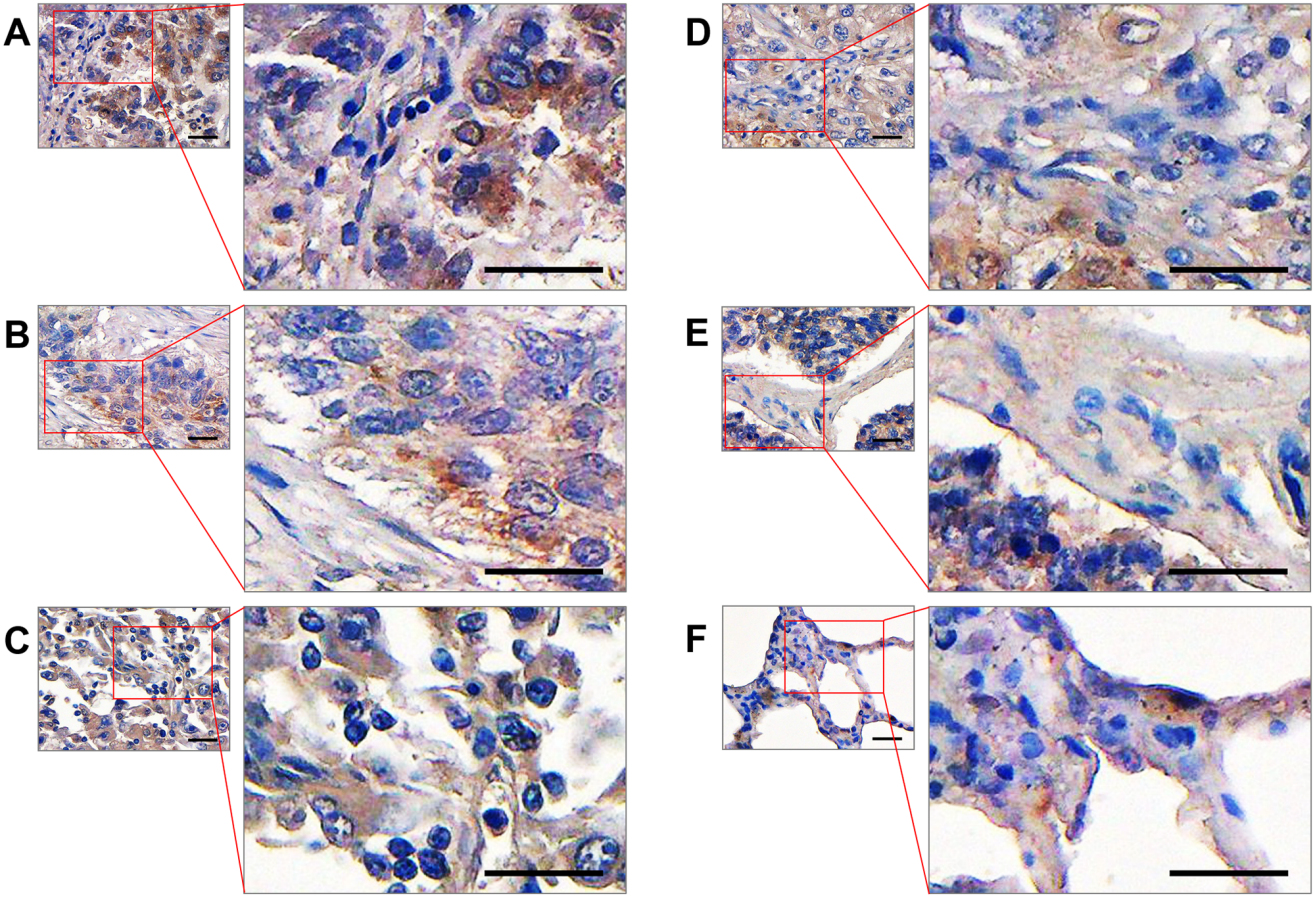
Immunohistochemical staining of ERK8 in lung cancer and normal lung tissue sections. A to F respectively present: papillary adenocarcinoma, squamous cell carcinoma, large cell carcinoma, adenosquamous carcinoma, atypical carcinoid, and normal lung tissue. Scale bar, 30 µm.

To further show that ERK8 can be recognized by our polyclonal antibody in human tissues, tissue array containing normal and cancer lung tissues were used for immunohistochemical staining. The results showed that our home-made anti-ERK8 antibody could detect ERK8 protein in human lung tissues, and the ERK8 protein was localized in both the nucleus and cytoplasm of lung cancer cells (Fig 7). Therefore, our polyclonal antibody against ERK8 can recognize endogenous ERK8 in tissues and would serve as an important and useful tool for further studies involving the detection of ERK8 protein at tissue levels.

## Discussion

Proposed to be a novel potential therapeutic target for cancer, ERK8 shows important roles in cell transformation, protection of genomic integrity, telomerase activity and autophagic process [7–10]. Interestingly, we have recently shown that ERK8 can be activated by arsenic trioxide and activated ERK8 subsequently promotes the phosphorylation and degradation of IκBα, leading to the activation of NF-κB and lung cancer cell apoptosis [15].

In order to further reveal the functions of ERK8, there is a need to have a highly sensitive and specific antibody against ERK8. However, we found that most of the commercial ERK8 antibodies cannot detect endogenous ERK8 protein and/or have low sensitivity and specificity, which may hinder further research for ERK8. In the present study, we presented the detailed protocol for the production and application of a highly sensitive and specific antibody against ERK8.

In our study, as a matter of fact, we have initially predicted two different antigenic region of human ERK8 protein by the B-cell epitopes prediction server (from region 348 to 419 (72 amino acids) and from region 446 to 473 (28 amino acids). Although we have successfully expressed these two GST-fusion proteins, after immunizing the rabbits, yet, we found that the only the anti-ERK8 antibody from rabbits immunized with the 28 aa-protein sequence can recognize the ERK8 protein while the 72 aa-protein sequence cannot (data not shown). As noted, the polypeptide antigen sequence we selected was located at the C-terminal region of ERK8, while most of commercial ERK8 antibodies were raised against the N-terminal region (i.e., with lower immunogenicity scores, Fig 1B); which may be the reason of our home-made anti-ERK8 antibody’s high performance over commercial ERK8 antibodies. Indeed, our home-made anti-ERK8 antibody can be extensively used to detect ERK8 protein in both native and denatured states as validated by immunoblots, ELISA, and immunofluorescence staining. Moreover, we verified the localization of ERK8 in both the nucleus and cytoplasm of lung cancer tissues by immunohistochemical analyses. Furthermore, the sensitivity and specificity of our purified polyclonal anti-ERK8 antibody which stored in 1×PBS containing 20% glycerol and 0.01% NaN_3_ remain relatively stable even after storing it at −80°C for one month and up to more than one year (S4 Fig). Thus, our study provide a valuable tool for further exploration of ERK8 at protein level and also the histological examination of ERK8 expression in normal and tumor tissues.

In conclusion, our home-made purified polyclonal anti-ERK8 antibody can be routinely used for the detection of both endogenous and ectopically-expressed ERK8 proteins in multiple biological applications, which we believe it will be a useful and reliable tool for further elucidation of the biological function of ERK8.

## Supporting information

S1 Fig Authenticity of GST-ERK8(28aa) protein by LC-MS/MS analysis. (A) SDS—PAGE gel stained with silver staining. Lane 1: protein ladder; Lane 2: purified GST-ERK8(28aa) protein (indicated by red arrow); (B) LC-MS/MS result of the excised band of GST-ERK8(28aa) protein.
(TIF)

S2 Fig Comparison of the efficacy of our purified anti-ERK8 antibody with four commercial ERK8 antibodies by immunofluorescence assay. Co-immunostaining of transient-transfected Xpress-ERK8 in HeLa cells detected by anti-Xpress antibody (red) and anti-ERK8 antibody (green). A to D show the results of four different commercial ERK8 antibodies and E show the results of our purified home-made anti-ERK8 antibody.
(TIF)

S3 Fig Only ERK8 but not RSK2 signal is co-localized with Xpress signal. The pcDNA4-Xpress-RSK2 or pcDNA4-Xpress-ERK8 plasmid was transient-transfected into HeLa cells and the cells were co-immunostained with anti-Xpress antibody (red) and our purified home-made anti-ERK8 antibody (green).
(TIF)

S4 Fig Stability of our polyclonal antibody against ERK8. Western blot result using purified polyclonal anti-ERK8 antibody stored at −80°C for one month (A) and more than one year (B). Lane 1: HeLa cells transfected with pcDNA3.1; Lane 2: HeLa cells transfected with pcDNA3.1-ERK8. Single asterisk indicates endogenous ERK8 while double asterisk indicates ectopically-expressed ERK8.
(TIF)
