# Supplementary figures and images for: Purification and characterization of a highly specific polyclonal antibody against human extracellular signal-regulated kinase 8 and its detection in lung cancer
Source: PLoS One. 2017 Sep 13;12(9):e0184755. doi: 10.1371/journal.pone.0184755 (PMC5597239; doi:10.1371/journal.pone.0184755)

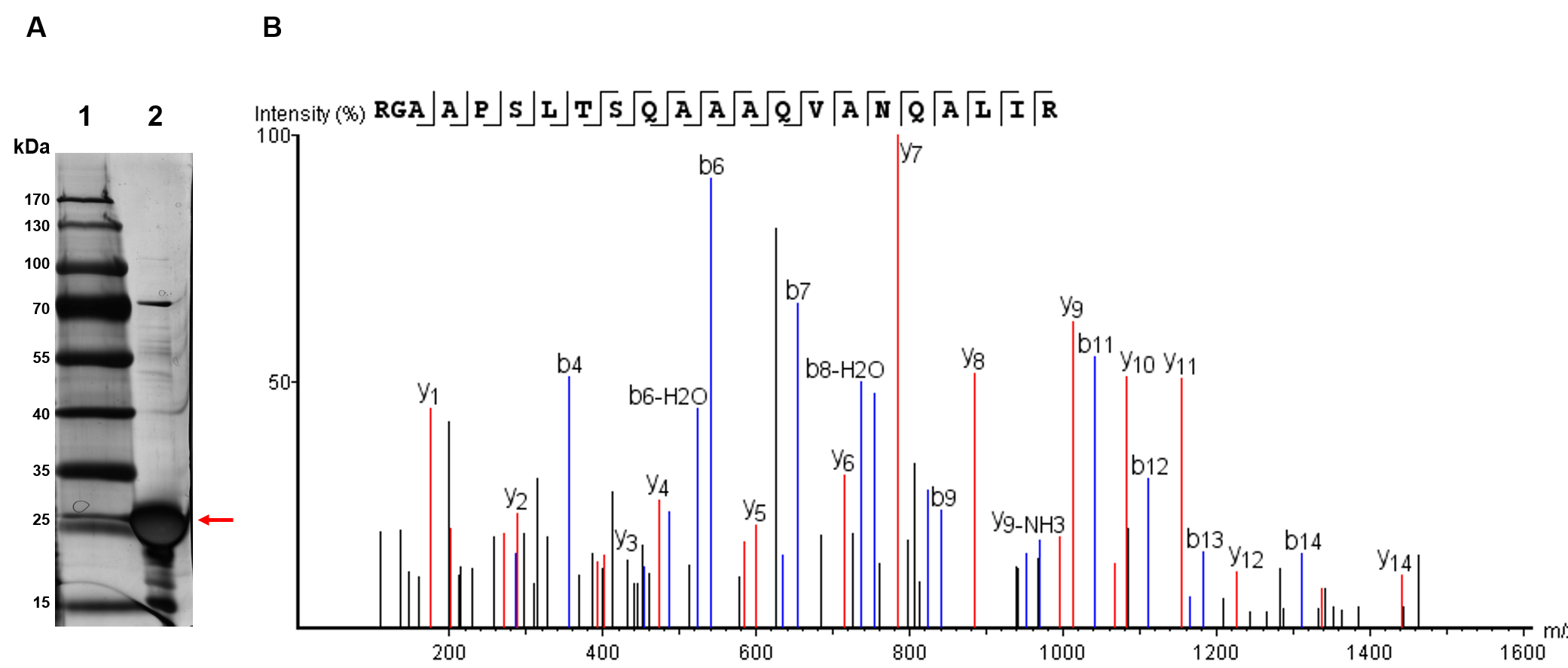

Supplement: S1 Fig — (A) SDS—PAGE gel stained with silver staining. Lane 1: protein ladder; Lane 2: purified GST-ERK8(28aa) protein (indicated by red arrow); (B) LC-MS/MS result of the excised band of GST-ERK8(28aa) protein. (TIF) [file pone.0184755.s001.tif]

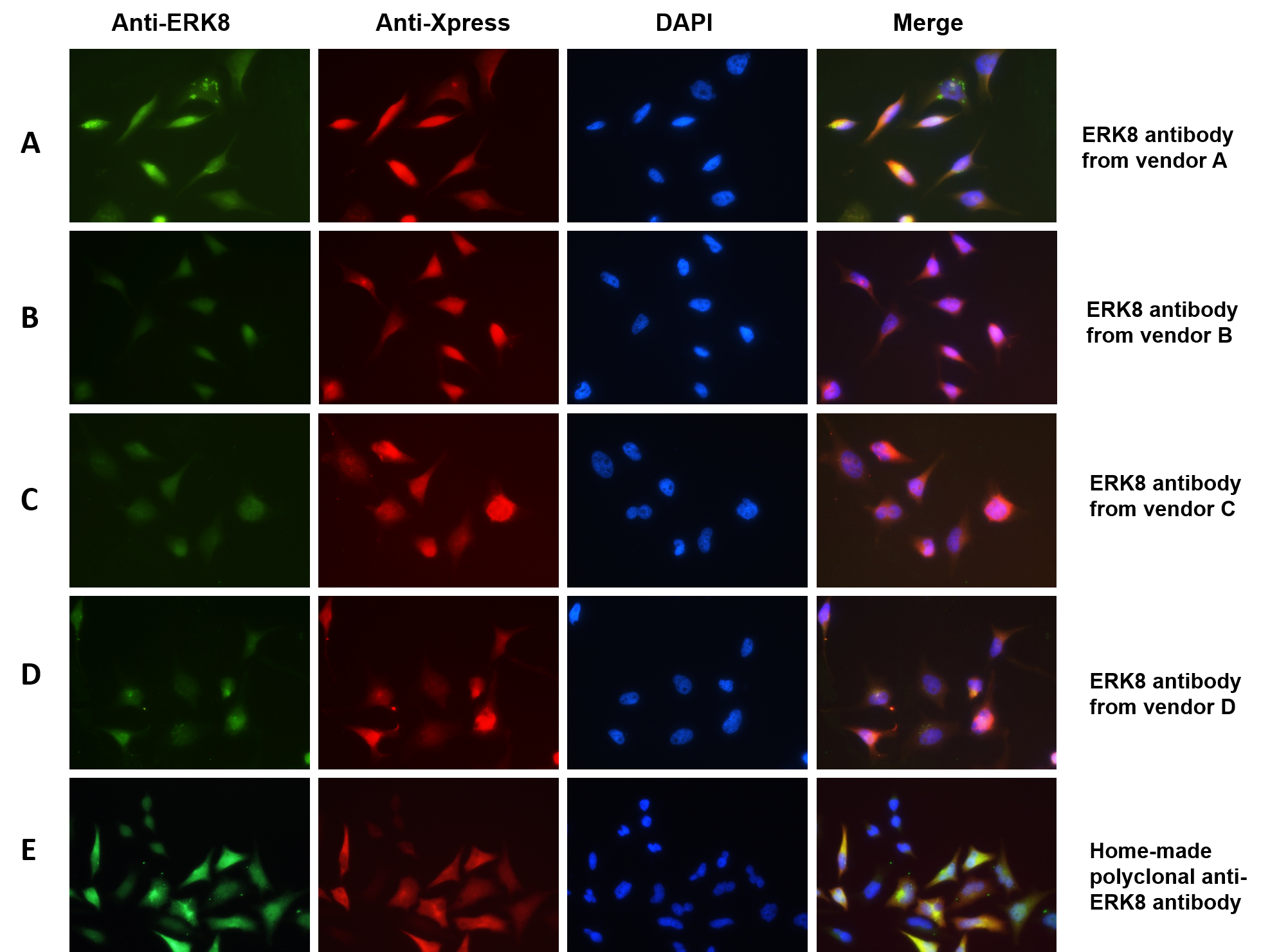

Supplement: S2 Fig — Co-immunostaining of transient-transfected Xpress-ERK8 in HeLa cells detected by anti-Xpress antibody (red) and anti-ERK8 antibody (green). A to D show the results of four different commercial ERK8 antibodies and E show the results of our purified home-made anti-ERK8 antibody. (TIF) [file pone.0184755.s002.tif]

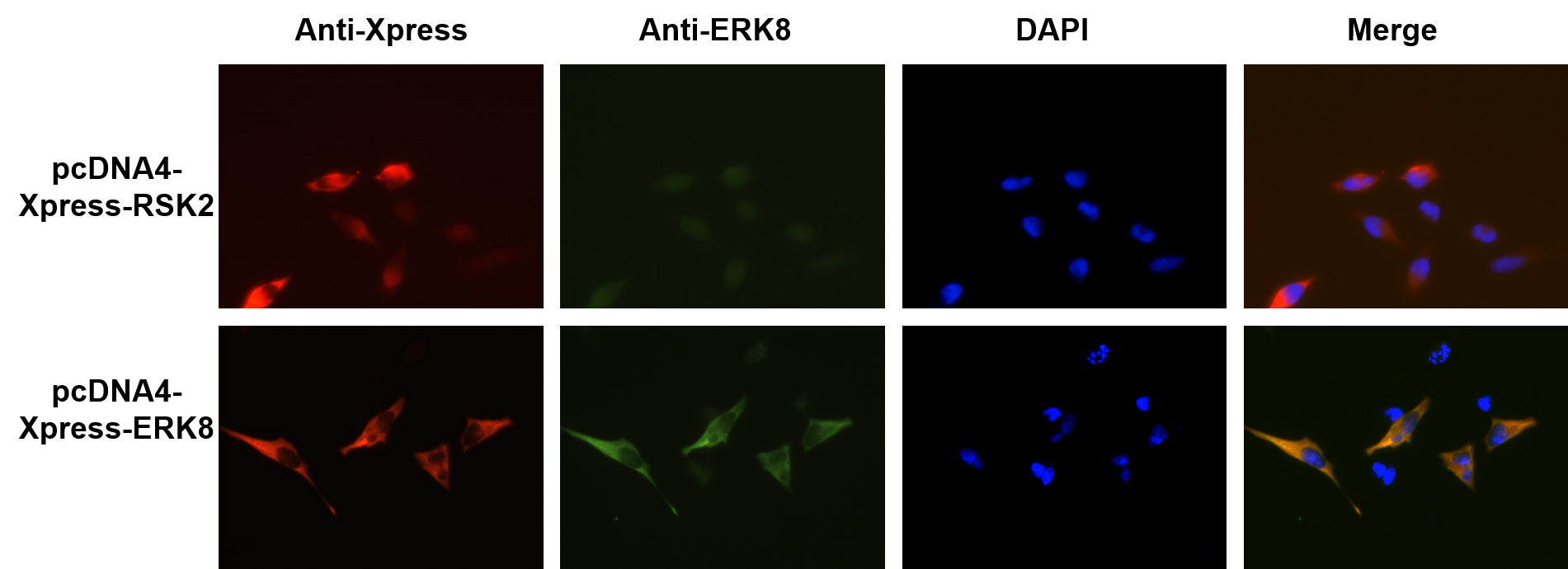

Supplement: S3 Fig — The pcDNA4-Xpress-RSK2 or pcDNA4-Xpress-ERK8 plasmid was transient-transfected into HeLa cells and the cells were co-immunostained with anti-Xpress antibody (red) and our purified home-made anti-ERK8 antibody (green). (TIF) [file pone.0184755.s003.tif]

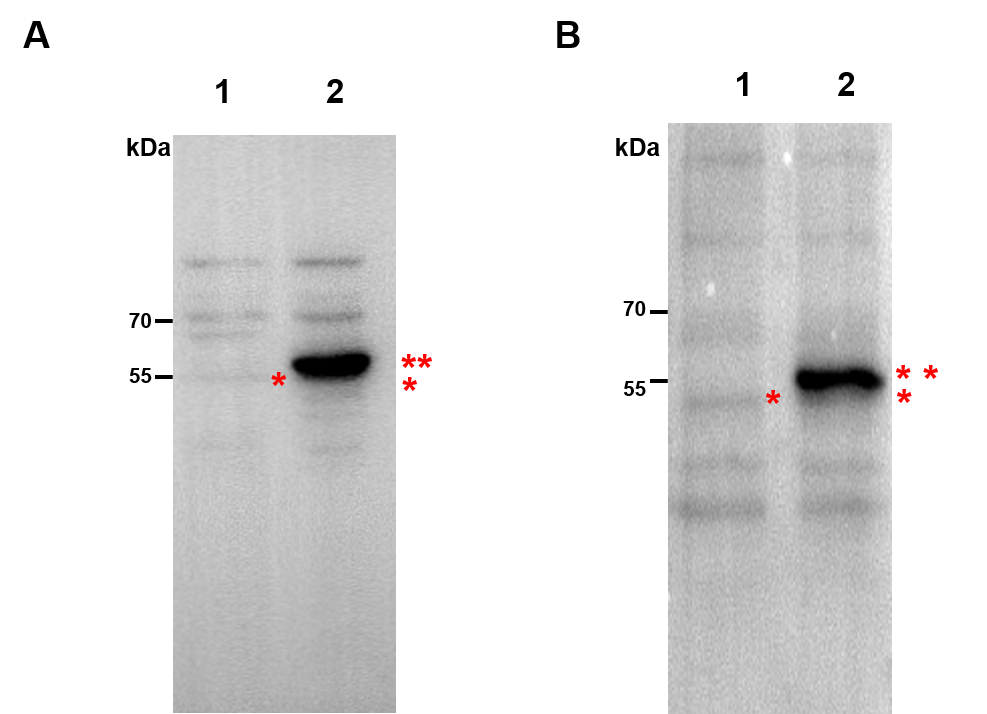

Supplement: S4 Fig — Western blot result using purified polyclonal anti-ERK8 antibody stored at −80°C for one month (A) and more than one year (B). Lane 1: HeLa cells transfected with pcDNA3.1; Lane 2: HeLa cells transfected with pcDNA3.1-ERK8. Single asterisk indicates endogenous ERK8 while double asterisk indicates ectopically-expressed ERK8. (TIF) [file pone.0184755.s004.tif]
